# Phylogenetic and functional niche breadth in two taxa of arthropod ectoparasites: correlations with ecomorphological traits are scale-dependent

**DOI:** 10.1007/s00436-026-08656-8

**Published:** 2026-03-03

**Authors:** Boris R. Krasnov, Vasily I. Grabovsky, Maxim V. Vinarski, Natalia P. Korallo-Vinarskaya

**Affiliations:** 1 Mitrani Department of Desert Ecology, Swiss Institute for Dryland Environmental and Energy Research, Jacob Blaustein Institutes for Desert Research, Ben-Gurion University of the Negev, Sede Boqer Campus, Midreshet Ben-Gurion, Beer-Sheva, 84990 Israel; 2 Wyler Department of Dryland Agriculture, French Associates Institute for Agriculture and Biotechnology of Drylands, Ben-Gurion University of the Negev, Sede Boqer Campus, Midreshet Ben-Gurion, Beer-Sheva, Israel; 3 Laboratory of Macroecology and Biogeography of Invertebrates, Saint-Petersburg State University, Saint-Petersburg, Russia; 4 Laboratory of Parasitic Arthropods, Zoological Institute of the Russian Academy of Sciences, Saint-Petersburg, Russia

**Keywords:** Abundance, Body size, Host specificity, Phylogeny, Traits

## Abstract

We studied the relationships between phylogenetic (PNB) and functional (FNB) niche breadth and abundance and body size in fleas and gamasid mites in multiple regions across the Palearctic. We asked whether (a) PNB or FNB correlated with abundance or body size; (b) the pattern of this correlation differed between the regional (within a region) and the continental (across regions) scales; and (c) the regional PNB and FNB of a parasite was affected by the phylogenetic and functional, respectively, diversity of all hosts in that region. The relationships between abundance and PNB or FNB were mainly manifested on the continental scale, but mostly not on the regional scale, being positive in both taxa. The effect of body size on PNB and FNB was found in only a few regions for fleas and in no regions for mites. On the contrary, continental PNB and FNB did not correlate with flea body size but decreased with an increase in mite body size. Regional PNB and FNB values of some, but not all, flea and mite species were positively associated with the phylogenetic and functional, respectively, diversity of hosts available in a region. We conclude that the relationships between the abundance and body size of an ectoparasite and its PNB or FNB are scale dependent. These relationships may differ from or be similar to the relationships between abundance or body size and niche breadth measured as the size of a host spectrum, depending on spatial scale and ectoparasite taxon.

**Supplementary Information:**

The online version contains supplementary material available at 10.1007/s00436-026-08656-8.

## Introduction

Host specificity is commonly recognized as a fundamental property of a parasite species. It reflects the number and composition of host species that a parasite can exploit, receiving a fitness benefit from this exploitation (Poulin et al. 2011). As such, host specificity represents a parasite’s diet niche breadth, with parasites characterised by a higher host specificity possessing a narrower niche. However, a host provides parasites with not only food resources but also with a place for living, mating, and reproducing. From this perspective, the host spectra of periodic haematophagous ectoparasites, such as fleas or parasitic gamasid mites (in contrast to permanent ectoparasites such as lice or temporary ectoparasites such as ixodid ticks and mosquitoes), include not only host species whose blood satisfies parasites’ nutritional requirements but also those who possess shelters with air temperatures and relative humidity levels that these parasites can tolerate. In fact, the majority of fleas and all ectoparasitic gamasid mites (a) spend substantial (if not the main) part of their lifetimes in hosts’ shelters (burrows or nests) where their preimaginal stages develop and (b) are highly sensitive to the microclimatic conditions in these shelters (Marshall 1981; Radovsky 1985). Consequently, these parasites’ host specificity reflects their niche breadth in a broader sense than merely diet.

Many traditional measurements of a parasite’s niche breadth (= host specificity) consider it as the diversity of hosts that a parasite exploits, and thus, they comprise various metrics of biological diversity adopted from general community ecology, such as host species richness or indices that take into account the relative abundances of host species in a parasite’s host spectrum (numerical or structural host specificity; Rohde and Rohde 2008; Poulin et al. 2011). However, the diversity of a host spectrum should consider not only the number of host species but also the difference or similarity between these species from the perspective of their evolutionary relationships (i.e., phylogeny) and their traits. Therefore, a measure of a parasite’s niche breadth (= inverse host specificity), analogously to measures of biological diversity, must include not only the number and composition of taxonomic units, such as species, but also the number and composition of phylogenetic (lineages) and functional units (traits or trait complexes) (Faith 1992; Schmera et al. 2023). These phylogenetic and functional facets of a parasite’s niche breadth represent phylogenetic and functional host specificity (Poulin et al. 2011; De La Torre et al. 2022). Attempts to include phylogenetic relationships between hosts in measurements of host specificity were made in the early 2000 s (Poulin and Mouillot 2003). These were done using a classification presented in the form of a Linnaean ranking since comprehensive phylogenetic trees for the majority of host taxa were not yet available. The subsequent availability of detailed molecular phylogenetic trees (e.g., Bininda-Emonds et al. 2007 for mammals) allowed calculating phylogenetic host specificity in more sophisticated ways (e.g., Park et al. 2018; Krasnov and Shenbrot 2025).

The development of the concepts of phylogenetic/functional host specificity has led to the question of whether the patterns of the relationships between these host specificity facets and parasite species’ ecological or morphological traits are similar to those of the traditional metrics of numerical/structural host specificity. For example, Krasnov et al. (2005) found a positive correlation between fleas’ geographical range size and their host spectra’s size. A similar pattern was later reported for fleas’ phylogenetic and functional host specificity (Krasnov and Shenbrot 2025). In contrast, Krasnov et al. (2008) reported that the numerical host specificity of fleas did not vary with latitude, whereas their functional host specificity decreased from south to north (Krasnov and Shenbrot 2025). This suggested that numerical, phylogenetic, and functional host specificity could be driven similarly by some factors, but differently by others.

Abundance is one parasite species trait that has often been studied in its relation to host specificity, to test two alternative hypotheses, namely Brown’s “resource breadth hypothesis” (1984) (the ability of species with broader niches to attain higher local abundances) and Poulin’s “trade-off hypothesis” (1998) (the trade-off between the number of host species exploited and the abundance achieved by parasites in these hosts is due to the high cost of adaptations to multiple host defence mechanisms). In different host-parasite associations, either local abundance appeared to be higher (e.g., Krasnov et al. 2004a) or lower (e.g., Poulin 1999; Barger and Esch 2002) in host generalists than in host specialists or no relationship between local abundance and host specificity was found (Morand and Guégan 2000). Host specificity in these studies was measured as the size of a parasite’s host spectrum. Similarly, the opposite abundance-host specificity patterns were reported in different host-parasite associations when host specificity was considered from a phylogenetic perspective (De Angeli Dutra et al. 2021; Park et al. 2025). Moreover, a comparison of the patterns of the relationship between abundance and numerical versus quasi-phylogenetic [using Poulin and Mouillot’s (2003) indices of host taxonomic distinctness] host specificity, in the same host-parasite association, demonstrated that these patterns were similar in some host-parasite associations (e.g., Krasnov et al. 2004a) and opposite in other host-parasite associations (Poulin 1999; Poulin and Mouillot 2004). In general, the relationship between a parasite’s abundance and its phylogenetic host specificity has rarely been studied, whereas, to the best of our knowledge, a parasite’s abundance has never been related to its functional host specificity.

Body size is another parasite trait that has been tested for its correlation with host specificity, albeit rarely. This was done for two taxa of ectoparasitic arthropods in which body size can be reliably measured, namely gamasid mites and fleas (Krasnov et al. 2013; Surkova et al. 2018). Both studies considered host specificity as the number of exploited hosts. In addition, Surkova et al. (2018) also measured flea host specificity as the average phylogenetic distinctness between all pairs of host species. The latter measure takes into account, to some extent, phylogenetic relationships between hosts but does not represent their true phylogenetic diversity. No body size-host specificity relationship was found in fleas for either numerical or “quasi-phylogenetic” host specificity, whereas larger gamasid mites appeared to be more host specific than smaller ones, although this was the case for facultatively, but not obligatorily, haematophagous species. The association between a parasite’s body size and its functional host specificity has never been studied.

Here, we investigated the relationships between phylogenetic and functional host specificity (as inverse phylogenetic and functional, respectively, niche breadth) and characteristic abundance and body size in fleas and gamasid mites parasitic on small mammals in different regions across the Palearctic. First, we tested whether abundant or larger flea or mite species are characterised by higher or lower phylogenetic or functional niche breadth. Second, we considered the relationships between abundance or body size and phylogenetic or functional niche breadth at regional (= within a region) and at continental (= across regions) scales and asked whether (a) the pattern of these relationships differed between scales and (b) the mean regional niche breadth of a flea or mite species correlates with its continental niche breadth. Finally, we investigated whether the regional phylogenetic and functional niche breadth of a flea or mite species is affected by the phylogenetic and functional, respectively, diversity of hosts available in that region.

## Materials and methods

### Data on fleas, gamasid mites, and their host species

We obtained data on fleas and gamasid mites parasitic on small mammalian hosts (Rodentia, Soricomorpha, and ochotonid Lagomorpha) from published surveys that reported records of a given flea or mite species on a host species across 51 and 28 Palearctic regions, respectively (see references in Krasnov et al. 2015 for fleas and Krasnov et al. 2009 for mites). Prior to the analyses, we excluded from the dataset (a) commensal ubiquitous rodents (*Rattus rattus*, *Rattus norvegicus*, and *Mus musculus*) and their characteristic ubiquitous fleas (*Xenopsylla cheopis* and *Nosopsyllus fasciatus*) and (b) all flea and mite species that were only recorded on one or two host species either within a region or across all regions. The resulting dataset included 160 flea and 47 mite species (Supplementary Tables S1 and S2, respectively) recorded on 136 and 67 host species, respectively, in 49 and 28Palearctic regions, respectively, (see maps in Supplementary Figures S1 and S2).

### Host phylogenies and traits

Phylogenetic trees of hosts exploited by either fleas or mites (separately) were built using the subsets of 1000 trees taken randomly from Upham et al.’s (2019) 10000 species-level birth-death tip-dated completed trees for 5911 mammal species. From each of these subsets (i.e., one subset for flea hosts and another for mite hosts), we constructed a consensus tree using the “consensus.edge” function of the “phytools” package (Revell 2012) implemented in the R Statistical Environment (R Core Team 2025). Then, we ultrametrized each of these consensus trees using the “force.ultrametric” function (with option method=”extend”) of “phytools”. The polytomies were resolved using the “fix.poly” function of the R package “RRphylo” (Castiglione et al. 2018).

Each host species was characterised by 13 traits that, presumably, could determine a host’s suitability for flea or mite parasitism and/or the probability of a host to encounter many flea or mite species (see, for example, Krasnov 2008 for fleas). These traits were adult body mass, relative brain mass, maximal longevity, litter size, generation length (average age of the current cohort’s parents in days), dispersal distance (average distance between the place of birth and the place of reproduction, in km), hibernation/torpor (yes or no), fossoriality (fossorial/ground dwelling or above-ground dwelling), habitat breadth (number of distinct suitable level 1 IUCN habitats), trophic level (herbivore, omnivore, or insectivore), foraging stratum (ground level, scansorial, or arboreal), activity cycle (nocturnal only, cathemeral/crepuscular, or diurnal only), and log-transformed geographic range size. Data on the majority of traits were taken from the PanTHERIA database (Jones et al. 2009), the EltonTraits database (Wilman et al. 2014), and the COMBINE database (Soria et al. 2021). Geographic range sizes were calculated from range maps (IUCN 2025).

### Flea phylogeny and mite taxonomy

As a backbone for flea phylogeny, we used the most comprehensive molecular phylogenetic tree (Zhu et al. 2015) that included the majority of flea genera, albeit not species, from our data. The topology of the genera and species that were not in Zhu et al.’s (2015) tree was established based on morphologically defined taxonomic positions (Hadfield et al. 2014). No information on branch lengths was available, so all branches were arbitrary assigned the length of 1 (Felsenstein 2004). Then, the tree was ultrametrized as described above.

No comprehensive molecular or morphological phylogeny of gamasid mites is available. Consequently, we used the hierarchical classification tree with three above-species levels (genus, subfamily, and family) as a proxy for mite phylogeny (see references for mite taxonomy in Krasnov et al. 2019). Then, a quasi-phylogenetic dendrogram for mites was constructed using the “hclust” function of the R package “stats”, which was then transformed into a quasi-phylogenetic tree using the “as.phylo.formula” function of the R package “ape” (Paradis and Schliep 2019).

### Calculation of phylogenetic and functional niche breadth

We constructed a presence/absence matrix with flea or mite species in rows and host species in columns for each region (regional matrices) and across all regions (continental matrices). Presence/absence data, rather than abundance and count data, are commonly used in parasite ecology because measurements of parasite occurrences are more reliable than measurements of abundances (Gotelli and Rohde 2002; see also Krasnov et al. 2021). The regional and continental phylogenetic/functional diversities of hosts exploited by a particular flea or mite species were considered as the regional and continental phylogenetic/functional niche breadth, respectively, of a given ectoparasite species. Calculations of phylogenetic and functional niche breadth followed the approaches proposed by Ricotta et al. (2016, 2018). In this approach, the functional diversity of an assemblage of hosts exploited by a flea or mite species is computed as Rao’s (1982) quadratic diversity (*Q*), by averaging pairwise between-species functional dissimilarities (see Ricotta et al. 2016 for details). Pairwise functional dissimilarity can take values from 0 to 1, being 0 if the two species are functionally identical and 1 if they are maximally dissimilar. We calculated the *Q* of a host assemblage for each flea species, using the “uniqueness” function of the R package “adiv” (Pavoine 2020) from the distance trait matrices of hosts that were constructed using the Gower distance coefficient with the “gowdis” function implemented in the R package “FD” (Laliberté and Legendre 2010; Laliberté et al. 2014). The Gower coefficient allows constructing a dissimilarity matrix from data composed of quantitative, categorical, ordinal, and binary variables (Gower 1971).

According to Ricotta et al. (2018) and similarly to functional diversity, phylogenetic diversity can be calculated from pairwise phylogenetic distances between species after rescaling them to a unit range. However, and in contrast to functional diversity, the calculation of phylogenetic diversity takes into account the branching pattern of a phylogenetic tree that represents the evolutionary relationships between species. For this, an ultrametric phylogenetic tree is segmented at each node, so that there are no internal nodes between each two segments (see Ricotta et al. 2018 for details). Then, age is assigned to each node, starting from the root node (maximal age) to the tips of a tree (zero age). As a result, two subsequent segments outline an evolutionary period, with each of these periods containing a number of lineages. At each node age, lineage diversity is calculated using a traditional diversity index *D*_*k*_ (in our case, species richness) under the assumption that all species are maximally and equally distinct. The diversities *D*_*k*_ over a certain evolutionary interval are averaged, which results in the assemblage-level phylogenetic diversity *D*_*P*_ that thus contains information on the branching pattern. We calculated *D*_*P*_ using the “treeUniqueness” function of the “*adiv*” package with the option “index=richness”.

### Data on the characteristic abundance and body sizes of fleas and mites

Data on the characteristic abundance and body sizes of flea and mite species were taken from our earlier studies (Krasnov et al. 2013, 2015; Korallo-Vinarskaya et al. 2013; Surkova et al. 2018). Abundances of the same flea and mite species on the same host species, but in different regions, appeared to be more similar to each other than expected by chance, varying significantly between, but not within, flea and mite species (Krasnov et al. 2006; Korallo-Vinarskaya et al. 2009). Therefore, the abundance of a given flea or mite species can be considered as a true species characteristic that varies only within relatively narrow species-specific boundaries. We focused on flea and mite abundances on their principal hosts, identified as the host species on which a flea or mite attains its highest abundance (see details in Krasnov et al. 2004b, 2013).

The body size of a flea was measured as a maximal body length (see details in Surkova et al. 2018), whereas the midline length of the dorsal shield was used as the indicator of mite body size (see details in Korallo-Vinasrkaya et al. 2013). This measurement was selected because the dorsal shield is the most heavily sclerotized part of a mite’s body that is not affected by engorgement. Both fleas and mites are characterised by female-based size sexual dimorphism. Consequently, we used the median of the average male and the average female body size as a single value that characterises the body size of a species.

### Data analyses

First, we estimated the repeatability of the phylogenetic and functional niche breadth of fleas and mites across regions to determine whether these facets of niche breadth are repeatable within flea or mite species that occurred in at least two regions (99 flea and 31 mite species). The repeatability of phylogenetic and functional niche breadth values was estimated using the intra-class correlation coefficient (ICC or R), which demonstrated the proportion of variation attributed to between-group (i.e., between-species in our case), as opposed to within-group (i.e., within-species in our case), variation. This was done using a linear mixed-effects model-based approach for Gaussian data with the R package “rptR” (Stoffel et al. 2017). Confidence intervals were estimated via 1000 bootstrap iterations.

Then, we tested the relationships between the phylogenetic and functional niche breadth of flea or mite species and their (a) characteristic abundance (response variable) and (b) body size (explanatory variable) in log-log space. This was done on both the regional (for the 38 and 16 regions in which at least eight flea or mite species were recorded), respectively, and the continental scale (for all flea and mite species). In addition, we tested whether the mean regional phylogenetic and functional niche breadth of a flea or mite species, recorded in at least two regions, correlated with its continental phylogenetic and functional niche breadth. Finally, we tested whether regional the phylogenetic and functional niche breadth was affected by the phylogenetic and functional diversity of hosts available in that region. For the latter analyses, we selected 17 flea and 13 mite species that were recorded in at least eight regions. In all analyses involving multiple flea or mite species, we controlled for the confounding effect of phylogenetic relationships between fleas or mites by applying phylogenetic generalized least squares (PGLS) models (Grafen 1989; Martins and Hansen 1997; Pagel 1999; Rohlf 2001; Freckleton et al. 2002) using the R package “caper” (Orme et al. 2025). Lambda, delta, and kappa values were optimized between bounds, using maximum likelihood. In the PGLSs, we used a phylogenetic tree for fleas and a quasi-phylogenetic (taxonomic) tree for mites (see above).

## Results

The repeatability analysis of the phylogenetic and functional niche breadth for 99 flea and 31 mite species, occurring in at least two regions, demonstrated that, although the within-species repeatability of the phylogenetic (measured as *D*_*P*_) and functional (measured as *Q*) niche breadth within a species was rather low (Table 1), it was, however, significant. The repeatability of both *Dp* and *Q* within mite species was lower than of that within flea species (Table 1).

**Table 1:** Values of the intra-class correlation coefficient (ICC), demonstrating the proportion of variation in the phylogenetic (*D*_*P*_) and functional (*Q*) niche breadth of flea and mite species attributed to between-species, as opposed to within-species, variation for 99 flea and 31 mite species. S.E.: standard error, CI: confidence interval

Taxon	Host specificity	ICC ± S.E.	2.5% CI	97.5% CI	*P*
Fleas	*D*_*P*_	0.14 ± 0.04	0.06	0.24	< 0.001
	*Q*	0.12 ± 0.04	0.04	0.20	< 0.001
Mites	*D*_*P*_	0.07 ± 0.05	0.00	0.17	0.01
	*Q*	0.06 ± 0.05	0.00	0.16	0.04

**Fig. 1:**
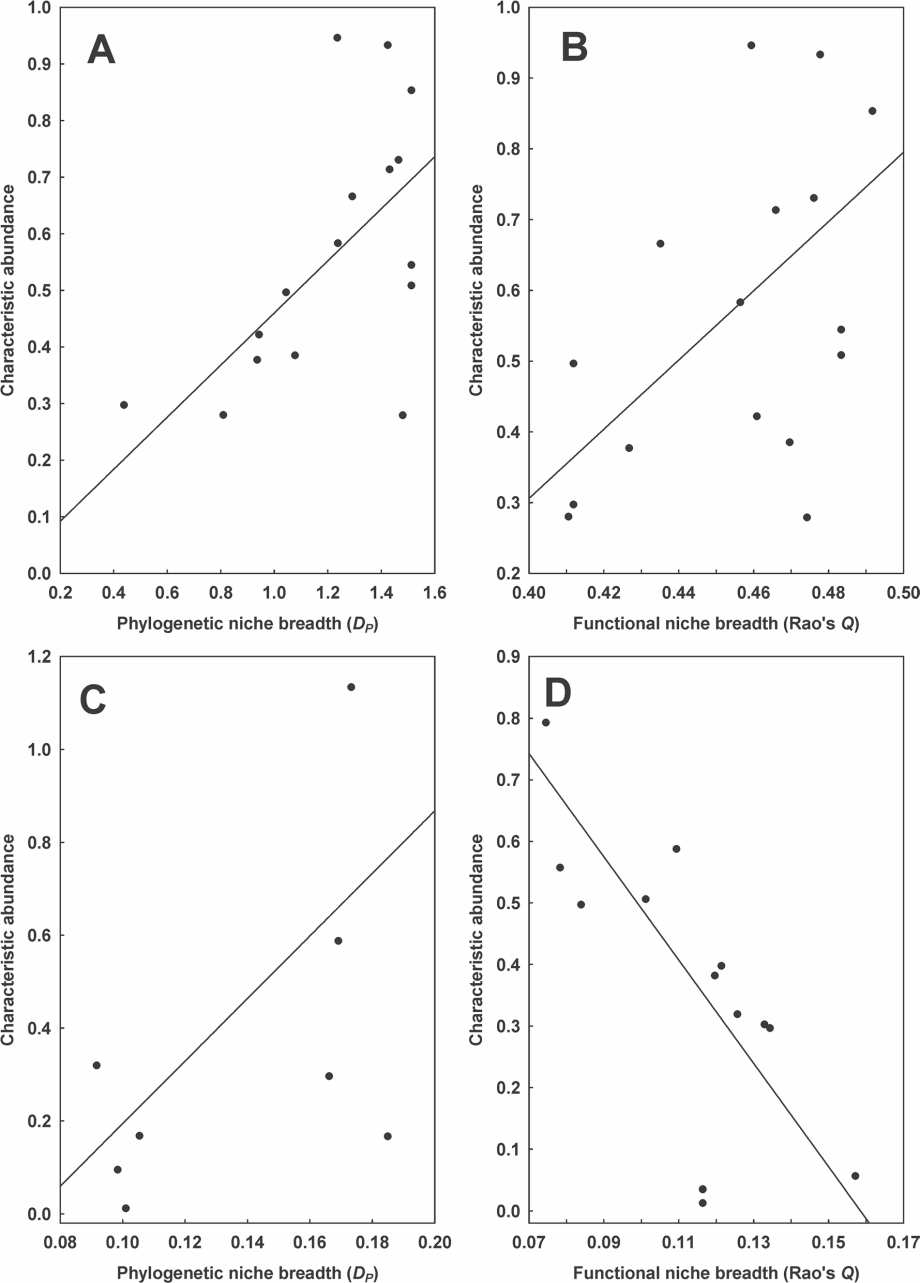
Relationships between the characteristic abundance and the regional (= within a region) phylogenetic (*D*_*P*_) niche breadth of fleas in Tyva (**A**) and mites in the Krasnodar region (**C**) and the functional (*Q*) niche breadth of fleas in Tyva (**B**) and mites in the Tomsk region (**D**) (in log-log space)

**Fig. 2:**
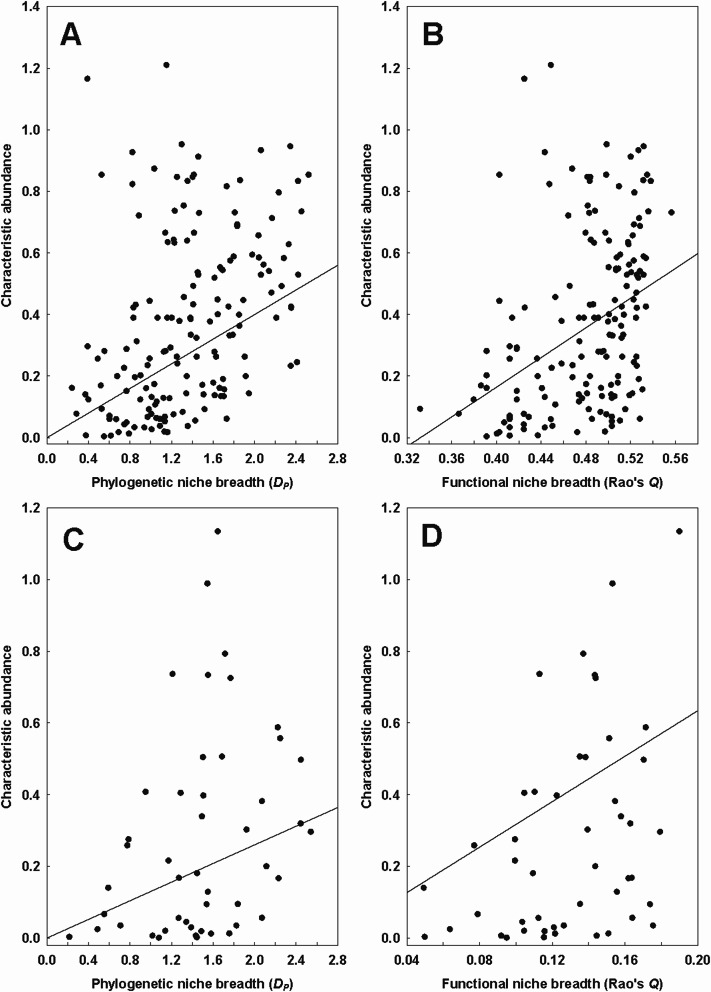
Relationships between the characteristic abundance and the continental (= across regions) phylogenetic (*D*_*P*_) and functional (*Q*) niche breadth of fleas (**A** and **B**, respectively) and mites (**C** and **D**, respectively) (in log-log space)

Significant relationships between phylogenetic niche breadth and characteristic abundance were found in only one of 38 regions for fleas and only one of 16 regions for mites (Table 2). Functional niche breadth correlated significantly with the characteristic abundance of fleas or mites in two of 38 regions and four of 16 regions, respectively (Table 2). In all of these regions, except the Tomsk region (Western Siberia), flea and mite species with broader phylogenetic and functional niche breadth were characterised by higher characteristic abundance (see illustrative examples for fleas in Tyva and mites in the Krasnodar region of European Russia in Figs. 1A-C). In contrast, characteristically abundant mites in the Tomsk region demonstrated a narrower functional niche breadth (Fig. 1D). On the continental scale, the characteristic abundance of both fleas and mites increased significantly with an increase in their phylogenetic and functional niche breadth (i.e., a decrease in phylogenetic and functional host specificity) (Table 3; Fig. 2).

**Table 2:** Summary of the phylogenetic generalized least squares (PGLS) models of the relationship between the regional phylogenetic (*D*_*P*_) or functional (*Q*) niche breadth of fleas and mites (explanatory variables) and their characteristic abundance (CA; response variable) after log-transformation. Shown only for regions in which the relationship between phylogenetic or functional niche breadth and characteristic abundance was found significant. *: *P* < 0.05, **: *P* < 0.01

Taxon	D_*P*_/Q	Region	Equation CA =	*R*^2^	F
Fleas	*D*_*P*_	Tyva	0.46 × *D*_*P*_ *	0.34	7.19
	*Q*	Kabardino-Balkaria	2.84 × Q*	0.39	5.70
		Tyva	−1.65 + 4.89 × Q*	0.28	5.53
Mites	*D*_*P*_	Buryatia	−0.58 + 6.74 × *D*_*P*_ *	0.31	6.80
	*Q*	Krasnodar region	0.15 × Q*	0.59	8.79
		Novosibirsk region	6.36 × Q*	0.34	5.15
		Oms region (forest-steppe zone)	8.38 × Q*	0.24	5.39
		Tomsk region	1.33–8.39 × Q**	0.60	15.11

**Table 3:** Summary of the phylogenetic generalized least squares (PGLS) models of the relationship between the continental phylogenetic (*D*_*P*_) or functional (*Q*) niche breadth of fleas and mites (explanatory variables) and their characteristic abundance (CA) response variable) after log-transformation. *: *P* < 0.05, **: *P* < 0.01

Taxon	D_*P*_/Q	Equation CA =	*R*^2^	F
Fleas	*D*_*P*_	0.20 × *D*_P_**	0.16	30.37
	*Q*	−0.80 + 2.41 × Q**	0.16	28.84
Mites	*D*_*P*_	0.13 × *D*_P_*	0.09	4.46
	*Q*	3.17 × Q**	0.22	12.36

**Fig. 3:**
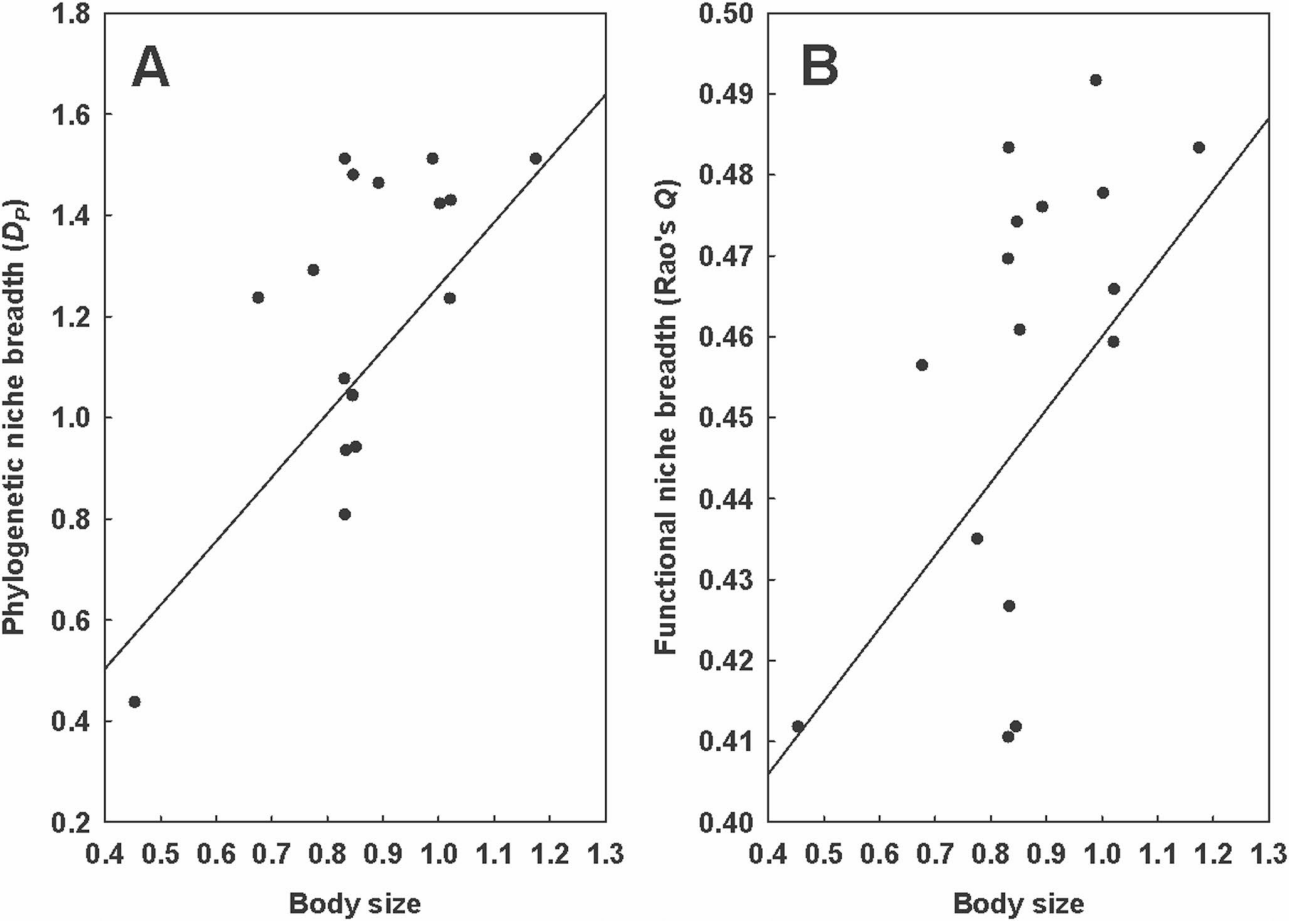
Relationships between the body size and the regional (= within a region) phylogenetic (*D*_*P*_; **A**) and functional (*Q*; **B**) niche breadth of fleas in Tyva (in log-log space)

**Fig. 4:**
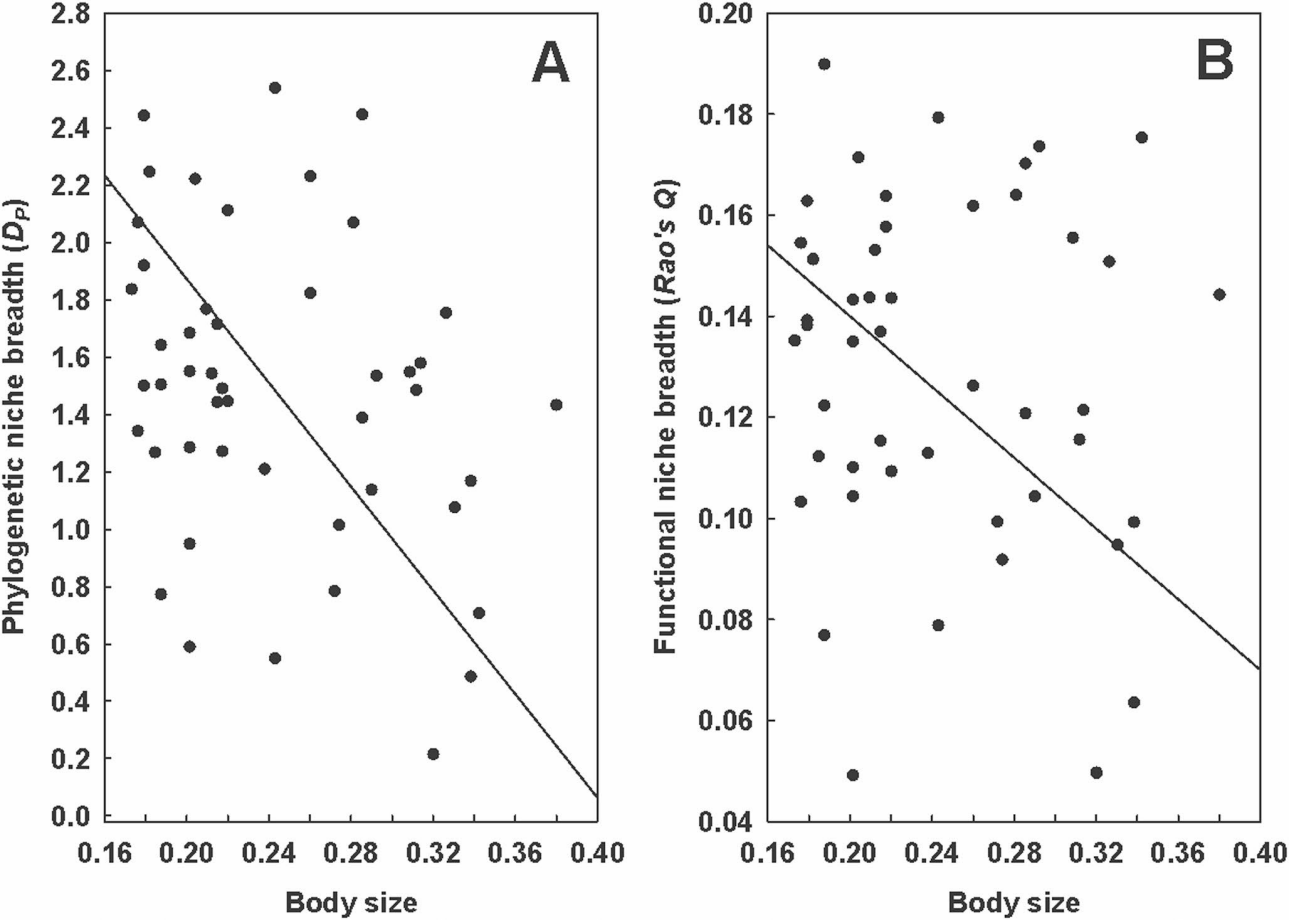
Relationships between the body size and the continental phylogenetic (*D*_*P*_; **A**) functional (*Q*; **B**) niche breadth of mites (in log-log space)

The regional phylogenetic and functional niche breadth of fleas correlated significantly with their body mass in three and two regions, respectively (Table 4; Fig. 3), whereas no relationship between either facet of regional niche breadth and mite body size was found in either region. On the continental scale, phylogenetic and functional niche breadth did not vary with flea body size, but both were narrower in larger mites (*D*_*P*_ = 3.68–9.04 × body size, *R*^*2*^ = 0.24, *F* = 14.02 and *Q* = 0.21–0.35 × body size, *R*^*2*^ = 0.08, *F* = 4.06; *P* < 0.05 for both) (Table 5; Fig. 4).

**Table 4:** Summary of the phylogenetic generalized least squares (PGLS) models of the relationship between the regional phylogenetic (*D*_*P*_) or functional (*Q*) niche breadth of fleas (response variables) and their body size (BS; explanatory variable) after log-transformation. Shown only for regions in which the relationship between phylogenetic or functional niche breadth and body size was found significant. *P* < 0.05 for all

D_*P*_/Q	Region	Equation D_*P*_ = or Q =	*R*^2^	F
*D*_*P*_	Tyva	1.26 × BS*	0.42	10.27
	Novosibirsk region	1.50 × BS*	0.27	6.69
	Terskey-Alatau range	1.31 × BS*	0.31	5.31
*Q*	Tyva	0.37 + 0.09 × BS*	0.31	6.42
	Novosibirsk region	0.34 + 0.12 × BS*	0.22	5.11

**Table 5:** Summary of the phylogenetic generalized least squares (PGLS) models of the relationship between the continental phylogenetic (*D*_*P*_) or functional (*Q*) niche breadth of fleas and mites (response variables) and their body size (BS; explanatory variable) after log-transformation. *: *P* < 0.05, **: *P* < 0.01

Taxon	D_*P*_/Q	Equation D_*P*_ = or Q =	*R*^2^	F
Fleas	*D*_*P*_	0.20 × BS**	0.16	30.37
	*Q*	−0.80 + 2.41 × BS**	0.16	28.84
Mites	*D*_*P*_	0.13 × BS*	0.09	4.46
	*Q*	3.17 × BS**	0.22	12.36

**Fig. 5:**
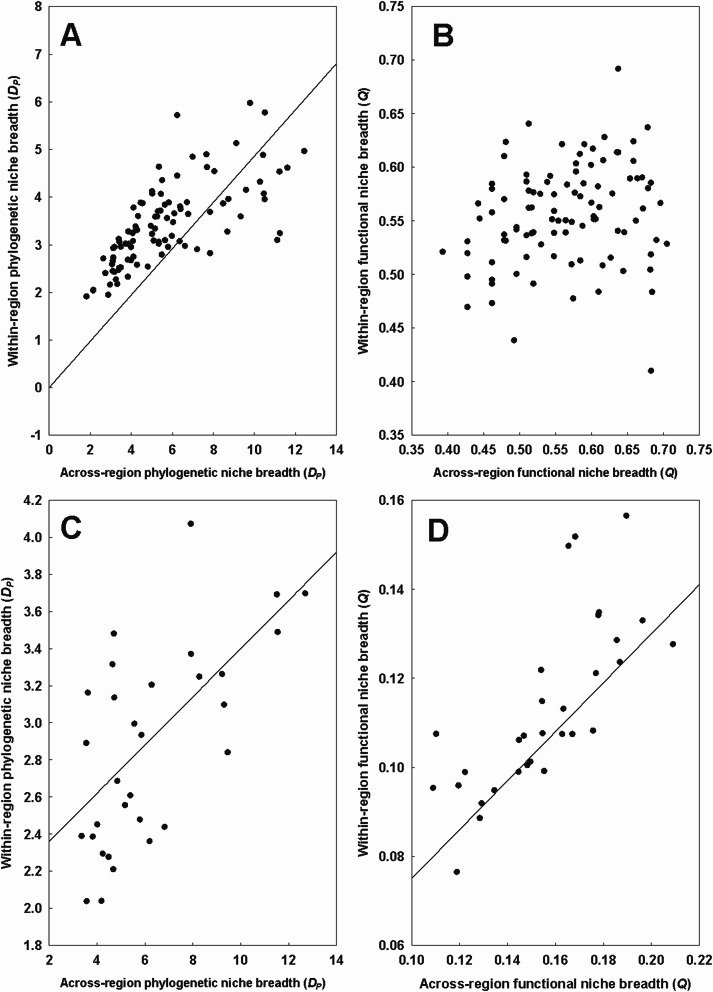
Relationships between the mean regional (= within a region) and the continental (= across regions) phylogenetic (*D*_*P*_) and functional (*Q*) niche breadth of fleas (**A**, **B**) and mites (**C**, **D**)

Regional phylogenetic niche breadth correlated positively with continental phylogenetic niche breadth in both fleas and mites (Table 6; Fig. 5A, C). This was also the case for functional niche breadth in mites, but not in fleas (Table 6; Fig. 5D, B).

**Table 6:** Summary of the phylogenetic generalized least squares (PGLS) models of the relationship between the regional (*r*; response variables) and continental (*c*; explanatory variables) phylogenetic (*D*_*P*_) or functional (*Q*) niche breadth of fleas and mites. *: *P* < 0.05, **: *P* < 0.01

Taxon	D_*P*_/Q	Equation D_*P*_-*r* = or Q-*r* =	*R*^2^	F
Fleas	*D*_*P*_*-r*	2.21 + 0.22 × *D*_*P*_-c**	0.50	95.87
	*Q*-*r*	Intercept only		
Mites	*D*_*P*_*-r*	2.10 + 0.13 × *D*_*P*_-c**	0.35	15.54
	*Q*-*r*	0.55 × Q-c**	0.52	32.11

**Fig. 6:**
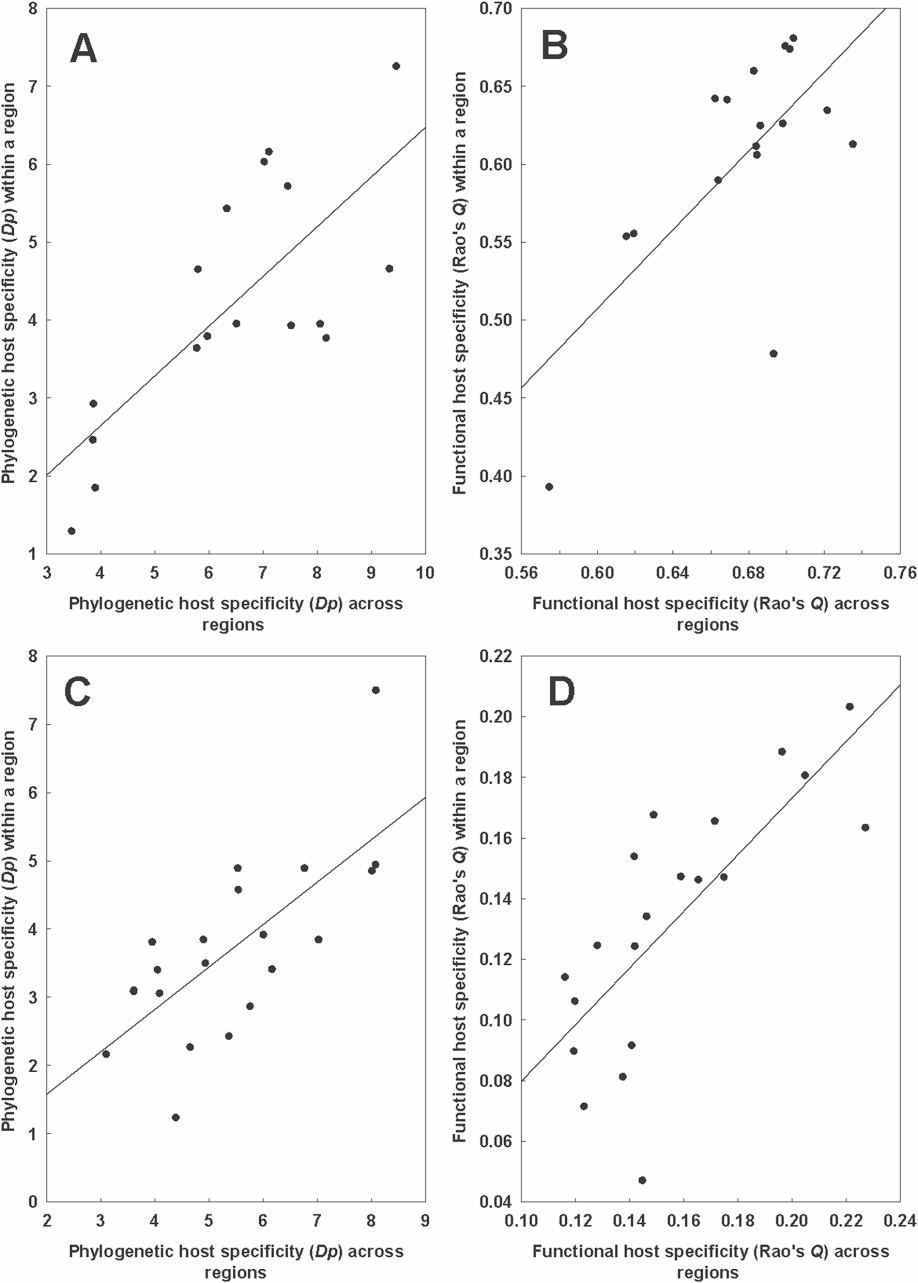
Relationships between the regional (= within a region) phylogenetic (**A**, **C**) and functional (**B**, **D**) niche breadth of a flea *Ctenophthalmus assimilis* (**A**, **B**) and a mite *Eulaelaps stabularis* (**C**, **D**) and the regional phylogenetic and functional diversity of all available hosts

Regional phylogenetic niche breadth was positively affected by the phylogenetic diversity of all hosts available in that region in six of 17 flea species and five of 13 mite species (Table 7). For functional niche breadth, this relationship was found in five flea and four mite species (Table 7). In four flea and four mite species, both patterns were found. Illustrative examples with a flea *Ctenophthalmus assimilis* and a mite *Eulaelaps stabularis* are presented in Fig. 6 (A-B and C-D, respectively).

**Table 7:** Summary of the phylogenetic generalized least squares (PGLS) models of the relationship between the log-transformed regional phylogenetic (*D*_*P*_) or functional (*Q*) niche breadth of a flea and a mite species (response variables) and the log-transformed phylogenetic (*D*_*P*_*h*) or functional (*Qh*) diversity, respectively, of available hosts in that region (explanatory variable). Only flea and mite species for which the relationship between phylogenetic or functional host specificity and phylogenetic or functional host diversity was found significant are shown. *: *P* < 0.05, **: *P* < 0.01

Taxon	D_*P*_/Q	Species	Equation	*R*^2^	F
Fleas	*Dp*	*Amalaraeus penicilliger*	0.47 × *D*_*P*_h **	0.35	11.99
		*Ctenophthalmus assimilis*	0.64 × *D*_*P*_h **	0.56	19.32
		*Hystrichopsylla talpae*	0.64 × *D*_*P*_h **	0.61	20.52
		*Megabothris rectangulatus*	0.50 × *D*_*P*_h *	0.49	12.65
		*Megabothris turbidus*	0.52 × *D*_*P*_h**	0.62	19.81
		*Palaeopsylla soricis*	0.52 × *D*_*P*_h**	0.48	12.86
	*Q*	*Ctenophthalmus assimilis*	1.26 × Qh**	0.47	13.23
		*Frontopsylla elata*	1.41 × Q*	0.37	8.87
		*Megabothris rectangulatus*	1.12 × Q**	0.48	12.24
		*Megabothris turbidus*	0.93 × Qh**	0.53	13.34
		*Palaeopsylla soricis*	0.92 × Qh**	0.34	7.20
Mites	*D*_*P*_	*Androlaelaps fahrenholzi*	0.79 × *D*_*P*_h*	0.24	4.62
		*Eulaelaps stabularis*	0.85 × *D*_*P*_h*	0.40	12.44
		*Haemogamasus ambulans*	0.73 × *D*_*P*_h**	0.41	14.13
		*Hirstionyssus eusoricis*	0.73 × *D*_*P*_h*	0.46	9.40
		*Hirstionyssus isabellinus*	0.70 × *D*_*P*_h**	0.35	11.83
	*Q*	*Androlaelaps fahrenholzi*	0.93 × Qh*	0.36	8.56
		*Eulaelaps stabularis*	1.19 × Qh**	0.43	14.41
		*Haemogamasus ambulans*	1.39 × Qh**	0.54	23.83
		*Hirstionyssus isabellinus*	0.73 × Qh*	0.26	7.64

## Discussion

We found positive relationships between flea or mite abundance and their phylogenetic and functional niche breadth on the continental scale, conforming to the “resource breadth” hypothesis (Brown 1984). The reason behind this may be that the same features that allow parasites to exploit hosts belonging to different phylogenetic lineages and possessing different traits, whatever these features might be, also allow them to attain higher characteristic abundance in their principal hosts. On the one hand, this resembles the pattern of the relationship between flea abundance and numerical host specificity reported earlier (Krasnov et al. 2004a). On the other hand, the latter study found this pattern on the regional scale, whereas either a positive or a negative relationship between abundance and phylogenetic or functional niche breadth on this scale was mostly absent. This suggests that highly abundant species (as a species-specific characteristic; Krasnov et al. 2006) have a general ability to become phylogenetic and/or functional host generalists due to, for example, a higher probability to encounter many hosts (Hurtado et al. 2024). However, this ability seems to be rarely realized on the regional scale.

The scale-dependence of the abundance-niche breadth relationship illustrates the difference between a fundamental and a realized niche (Hutchinson 1957). According to classical Hutchinsonian (1957) theory, a realized niche is narrower than a fundamental niche because it reflects the actual set of environmental conditions that a species occupies in nature, given actual biotic interactions and dispersal limitations. In the case of parasites, their realized niche within a region is determined, first and foremost, by the set of available hosts, from which a flea or a mite is forced to select those that possess traits allowing their exploitation (e.g., Krasnov et al. 2016 for fleas). Furthermore, the traits that allow a parasite to exploit a host can be similar in either phylogenetically close or phylogenetically distant (i.e., burrowing habits; Rodrigues et al. 2023) species. This likely results in interspecific variation in the phylogenetic diversity and/or functional diversity of a set of hosts selected by parasites in a region. Moreover, our results demonstrated that the phylogenetic and/or functional diversity of a set of hosts selected by a flea or a mite in a region is not necessarily associated with the phylogenetic and/or functional diversity of all hosts occurring in that region (see also Krasnov et al. 2004c). This can be associated with the ability of fleas and mites to shift their principal hosts across regions (e.g., Shenbrot et al. 2007 for fleas). The “replacement” hosts are not necessarily close relatives of the original hosts, and they may also belong to different phylogenetic lineages. The latter may happen due to the process known as “ecological fitting” (Janzen 1985), when a parasite’s primary requirement is the resource acquired from a host (e.g., blood composition for haematophages), rather than the natural “representation” of this resource (a given host species), with this resource being present in a number of host species (whether they are phylogenetically related or not). Consequently, a parasite may track the resource rather than a given host and, therefore, may switch to a new host offering this resource, independently of phylogenetic relatedness between this new host and the parasite’s original host (Brooks et al. 2006; D’Bastiani et al. 2023). Such a parasite can thus be considered as a phylogenetic host generalist on a large spatial scale but not necessarily a phylogenetic host specialist on the smaller scale. This scenario suggests that such a parasite has to be a functional host generalist at both scales, and given the link between higher abundance and a higher probability to encounter many hosts, the positive association between characteristic abundance and functional niche breadth is expected to occur on both regional and continental scales. However, this was not generally the case on the regional scale. Some reasons for this could be (a) that a parasite may respond to a few host traits only (the most important for them) rather than to trait complexes, whereas calculations of functional niche breadth consider multiple host traits simultaneously, and (b) that different parasite species respond to different host traits.

The positive association between a parasite’s characteristic abundance (i.e., attained in its principal host) and its phylogenetic niche breadth on a continental scale, found in our study, for haematophagous arthropods appeared not to be the case for other parasite taxa. For example, Poulin and Mouillot (2004) studied abundance and “quasi-phylogenetic” niche breadth in helminths infecting birds, using between-host taxonomic distances as a substitute for their phylogenetic relatedness. They found a negative correlation between mean abundance and the average taxonomic distance among host species in nematodes but a positive correlation between these variables in trematodes. Park et al. (2025) reported a negative association between abundance and phylogenetic niche breadth in helminths parasitic on mammals from several orders, but only when the abundance in the principal host species was considered. However, when parasite abundance was summed across all observed host species (total abundance), it was not associated with the phylogenetic relatedness of these hosts. De Angeli Dutra et al. (2021) demonstrated that the host phylogenetic range of avian haemosporidians was positively associated with their geographic ranges, which, in turn, correlated negatively with their local abundance, suggesting a negative relationship between host-associated phylogenetic niche breadth and abundance. However, this pattern did not appear to be universal and was mostly true for *Plasmodium* and not *Haemoproteus* lineages and in South America but not Europe. In general, the results of De Anglei Dutra et al. (2021) support our results on the scale-dependence of the relationship between parasite abundance and phylogenetic niche breadth.

Similarly to the relationships between abundance and niche breadth, those between flea or mite body size and the breadth of their phylogenetic and functional niches appeared to be scale dependent. Moreover, both the scale dependence and the pattern of the effect of body size on niche breadth differed between fleas and mites. In only a few regions and with the exception of Adzharia, larger fleas tended to be host generalists from both phylogenetic and functional perspectives, while no correlation between niche and body size was detected on the continental scale, supporting the results of Surkova et al. (2018) for numerical host specificity in these parasites. On the contrary, mite body size was found to be associated with their phylogenetic and functional niches only on the continental scale, with larger mites tending to be phylogenetic and functional host specialists, supporting the results of Krasnov et al. (2013) for numerical host specificity in facultatively hematophagous gamasids.

Negative relationships between body size and phylogenetic and functional, as well as numerical (Krasnov et al. 2013), niche breadth in mites can result from the presumably higher tolerance of smaller mites to microclimatic conditions in hosts’ burrows/nests, where mites spend almost their entire lives. As a result, smaller mites are thought to be able to reside in the shelters of multiple phylogenetically and functionally diverse hosts, whereas larger mites are sensitive to air temperature and humidity, so they choose only a restricted set of hosts whose shelters present mites with a suitable microclimate (Krasnov et al. 2013). In addition, mite body size has been shown to correlate negatively with their abundance (Krasnov et al. 2013), which, in turn, correlates positively with phylogenetic and functional niche breadth (this study). Consequently, the negative body size-niche breadth pattern could also be a consequence of the negative association between abundance and body size, coupled with the positive association between abundance and niche breadth. However, no relationship between body size and niche breadth was found in fleas, although their body size was previously shown to correlate positively with their abundance (Surkova et al. 2018). It is thus possible that the relationships between (a) abundance and body size and (b) abundance and niche breadth result in the relationship between body size and niche breadth in some, but not other, parasite taxa, depending on the taxon’s life history patterns or on the mediation of other, still unknown, factors or both.

In conclusion, different facets of parasite niche breadth (the size of host spectra, the phylogenetic and functional diversity of these hosts) could be similarly affected by some factors and differently by other factors. The relationships between the ecomorphological traits of parasites and their numerical, phylogenetic, and functional niche breadth differ in dependence on spatial scale and parasite taxa. The results of this study can help to predict abundance of ectoparasites causing disease outbreaks.

## Supplementary Information

Below is the link to the electronic supplementary material.

Supplementary Material 1 (DOCX 426 KB)

## Data Availability

Data on fleas and their hosts can be found in Hadfield et al. (2013) deposited in the Dryad repository. Part of the data on mites and their hosts can be found in Krasnov (2025) and deposited in the Mendeley Data repository. The remaining data on mites can be obtained from the corresponding author upon request.
