# Supplementary material for: Phylogenetic and functional niche breadth in two taxa of arthropod ectoparasites: correlations with ecomorphological traits are scale-dependent
Source: Parasitol Res. 2026 Mar 3;125(1):34. doi: 10.1007/s00436-026-08656-8 (PMC12957141; doi:10.1007/s00436-026-08656-8)
Supplement: Supplementary file 1 — Supplementary Material 1 (DOCX 426 KB) [file 436_2026_8656_MOESM1_ESM.docx]

**Supplementary Figures**

**
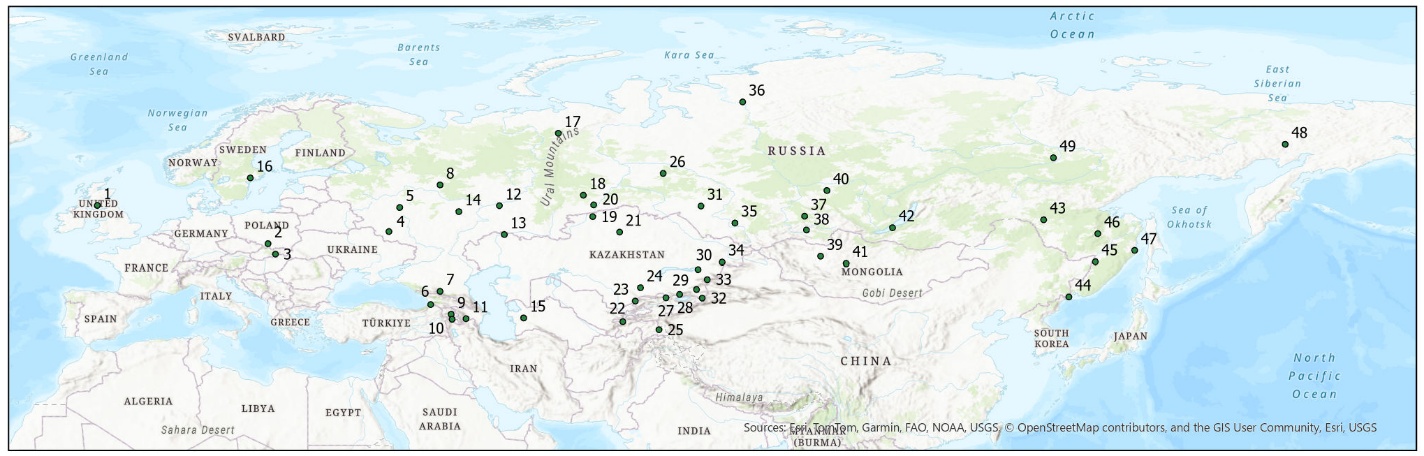
**

**Supplementary Fig. S1.** Regions where fleas were sampled.

1: Scotland, 2: Poland, 3: Slovakia, 4: Kursk region, 5: Moscow region, 6: Adzharia, 7: Kabardino-Balkaria, 8: Kostroma region, 9: Armenia, 10: Nakhichevan, 11: Southwestern Azerbaijan, 12: Volga-Kama region, 13: Ural River Valley, 14: Atyrau region, 15: Turkmenistan, 16: Sugaty, 17: Polar Ural Mountains, 18: Middle Ural Mountains, 19: Kostanay region, 20: Kurgan region, 21: Akmola region, 22: Hisar Range, 23 - Shimkent region, 24: Moiyunkum Desert, 25: Eastern Pamir Mountains, 26: Tomsk region, 27: North Kyrgyzstan, 28: Trans-Ili Alatau Range, 29: Sweden, 30: Eastern Balkhash Lake, 31: Novosibirsk region, 32: Terskey Alatau Range, 33: Dzhungarian Alatau Range, 34: Tarbagatai Mountains, 35: Altai Mountains, 36: Taymyr Peninsula, 37: Western Sayan Range, 38: Tyva, 39: Western Khangai Mountains, 40: Krasnojarsk region, 41: Central Khangai Mountains, 42: Selenga River Valley, 43: Amur River Valley, 44: Khasan Lake, 45: Ussury River Valley, 46: Bureya River Valley, 47: Khabarovsk region, 48: Northern Russian Far East, 49: Central Yakutia.

**
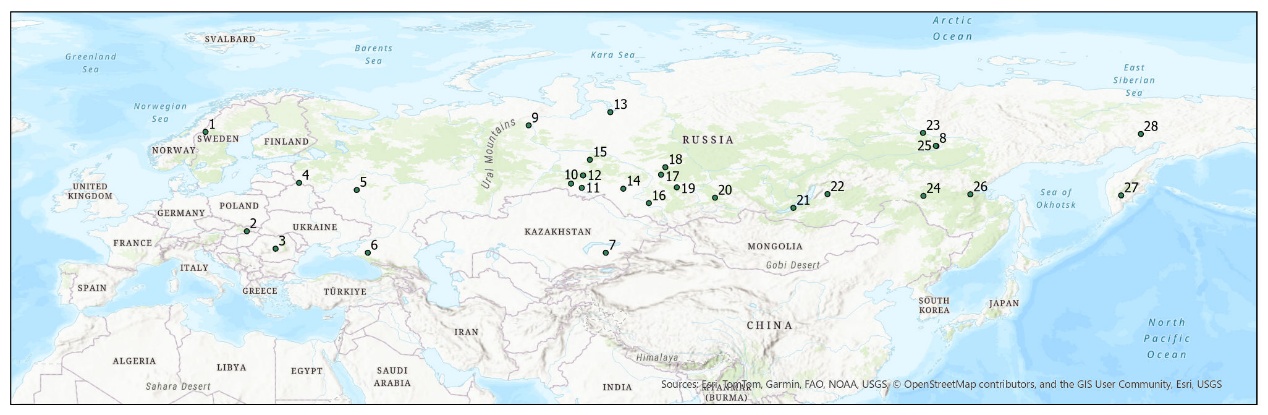
**

**Supplementary Fig. S2.** Regions where gamasid mite were sampled.

1: Norway, 2: Slovakia, 3: Romania, 4: Pskov region, 5: Moscow region, 6: Krasnodar region, 7: Balkhash Lake, 8: Kenkeme River Valley, 9: Ob River floodplain, 10: Tenis Lake, 11: Omsk region (forest-steppe zone), 12: Omsk region (forest zone), 13: Pur River Valley, 14: Novosibirsk region, 15: Tomsk region, 16: Altai Mountains, 17: Chulym River Valley, 18: Ket River Valley, 19: Kuznetsk Alatau range, 20: Western Sayan range, 21: Selenga River Valley, 22: Buryatia (Bu), 23: Western Predverkhoyan'ye, 24: Eastern Baikalo-Amur railway region, 25: Central Yakutia, 26: Southern Russian Far East, 27: Kamchatka Peninsula, 28: Northern Russian Far East.

**Supplementary Tables**

**Supplementary Table S1**

List of flea species that exploited at least three host species within a region

| Flea species | Mean (±S.E.) number of host species within a region | Total number of host species across regions | Number of regions where a flea species was recorded |
| --- | --- | --- | --- |
| *Amalaraeus arvicolae* | 5.0±0.0 | 10 | 2 |
| *Amalaraeus dissimilis* | 4.0±0.0 | 8 | 2 |
| *Amalaraeus penicilliger* | 8.2±1.7 | 206 | 25 |
| *Amphalius runatus* | 5.0±1.5 | 15 | 3 |
| *Amphipsylla anceps* | 4.5±0.9 | 18 | 4 |
| *Amphipsylla asiatica* | 4.0±0.0 | 4 | 1 |
| *Amphipsylla dumalis* | 5.0±1.0 | 10 | 2 |
| *Amphipsylla kuznetzovi* | 6±2.3 | 30 | 5 |
| *Amphipsylla longispina* | 7.3±1.8 | 22 | 3 |
| *Amphipsylla marikovskii* | 3.5±0.5 | 7 | 2 |
| *Amphipsylla montana* | 3. 7±0.3 | 11 | 3 |
| *Amphipsylla parthiana* | 3.0±0.0 | 6 | 2 |
| *Amphipsylla phaiomydis* | 4.0±0.0 | 8 | 2 |
| *Amphipsylla prima* | 4.0±0.0 | 4 | 1 |
| *Amphipsylla primaris* | 8.6±3.2 | 60 | 7 |
| *Amphipsylla rossica* | 6.7±1.6 | 100 | 15 |
| *Amphipsylla schelkovnikovi* | 5.0±1.5 | 15 | 3 |
| *Amphipsylla sibirica* | 8.4±2.3 | 59 | 7 |
| *Amphipsylla vinogradovi* | 13.0±5.4 | 52 | 4 |
| *Atyphloceras nuperus* | 4.0±0.0 | 8 | 2 |
| *Callopsylla caspia* | 5.5±1.8 | 44 | 8 |
| *Callopsylla saxatilis* | 3.0±0.0 | 6 | 2 |
| *Catallagia dacenkoi* | 6.1±1.9 | 43 | 7 |
| *Catallagia ioffi* | 4.6±0.8 | 32 | 7 |
| *Ceratophyllus anisus* | 3.0±0.0 | 3 | 1 |
| *Ceratophyllus indages* | 5.1±1.2 | 41 | 8 |
| *Ceratophyllus sciurorum* | 7.0±2.2 | 35 | 5 |
| *Citellophilus lebedewi* | 4.0±0.0 | 8 | 2 |
| *Citellophilus tesquorum* | 9.1±3.1 | 82 | 9 |
| *Citellophilus transcaucasicus* | 4.5±0.5 | 9 | 2 |
| *Citellophilus trispinus* | 6.0±1.6 | 30 | 5 |
| *Citellophilus ullus* | 4.7±1.2 | 14 | 3 |
| *Coptopsylla bairamalensis* | 8.0±0.0 | 16 | 2 |
| *Coptopsylla lamellifer* | 7.7±1.7 | 31 | 4 |
| *Coptopsylla olgae* | 8.0±0.0 | 16 | 2 |
| *Corrodopsylla birulai* | 8.7±2.2 | 122 | 14 |
| *Ctenophthalmus acuminatus* | 5.0±1.0 | 10 | 2 |
| *Ctenophthalmus agyrtes* | 12.1±1.9 | 97 | 8 |
| *Ctenophthalmus arvalis* | 9.4±2.9 | 47 | 5 |
| *Ctenophthalmus assimilis* | 11.7±2.3 | 210 | 18 |
| *Ctenophthalmus bisoctodentatus* | 5.3±1.2 | 32 | 6 |
| *Ctenophthalmus bogatschevi* | 3.5±0.5 | 7 | 2 |
| *Ctenophthalmus breviatus* | 10.4±2.8 | 52 | 5 |
| *Ctenophthalmus congener* | 8.0±0.0 | 16 | 2 |
| *Ctenophthalmus congeneroides* | 4.2±0.8 | 25 | 6 |
| *Ctenophthalmus dilatatus* | 4.0±0.0 | 4 | 1 |
| *Ctenophthalmus dolichus* | 8.3±2.0 | 25 | 3 |
| *Ctenophthalmus golovi* | 4.0±1.0 | 8 | 2 |
| *Ctenophthalmus hypanis* | 8.0±0.0 | 16 | 2 |
| *Ctenophthalmus inornatus* | 5.0±0.0 | 10 | 2 |
| *Ctenophthalmus nobilis* | 5.0±0.0 | 10 | 2 |
| *Ctenophthalmus obtusus* | 3.5±0.5 | 7 | 2 |
| *Ctenophthalmus orientalis* | 7.5±2.2 | 30 | 4 |
| *Ctenophthalmus pisticus* | 4.0±0.6 | 16 | 4 |
| *Ctenophthalmus proximus* | 6.0±1.8 | 24 | 4 |
| *Ctenophthalmus secundus* | 5.7±0.8 | 23 | 4 |
| *Ctenophthalmus shovi* | 6.0±0.0 | 12 | 2 |
| *Ctenophthalmus solutus* | 8.0±2.5 | 24 | 3 |
| *Ctenophthalmus teres* | 6.5±0.5 | 13 | 2 |
| *Ctenophthalmus uncinatus* | 11.6±2.5 | 93 | 8 |
| *Ctenophthalmus wagneri* | 13.2±3.9 | 66 | 5 |
| *Ctenophyllus armatus* | 3.0±0.0 | 3 | 1 |
| *Ctenophyllus subarmatus* | 3.0±0.0 | 10 | 2 |
| *Desertopsylla rothschildi* | 5.0±0.0 | 8 | 2 |
| *Doratopsylla dasycnema* | 9.0±1.9 | 57 | 8 |
| *Echidnophaga oschanini* | 6.4±1.8 | 39 | 5 |
| *Frontopsylla ambigua* | 4.0±0.0 | 17 | 2 |
| *Frontopsylla elata* | 7.1±2.0 | 91 | 18 |
| *Frontopsylla elatoides* | 8.0±3.3 | 55 | 4 |
| *Frontopsylla hetera* | 9.7±2.1 | 41 | 4 |
| *Frontopsylla luculenta* | 5.2±1.9 | 25 | 4 |
| *Frontopsylla macrophthalma* | 4.5±0.5 | 15 | 2 |
| *Frontopsylla ornata* | 7.0±2.2 | 20 | 4 |
| *Frontopsylla protera* | 8.3±3.2 | 24 | 3 |
| *Frontopsylla semura* | 6.0±2.0 | 18 | 2 |
| *Hystrichopsylla microti* | 4.5±0.9 | 19 | 4 |
| *Hystrichopsylla orientalis* | 10.0±2.1 | 24 | 3 |
| *Hystrichopsylla satunini* | 3.0±0.0 | 16 | 2 |
| *Hystrichopsylla talpae* | 10.4±1.9 | 134 | 16 |
| *Leptopsylla nana* | 4.0±0.8 | 48 | 5 |
| *Leptopsylla segnis* | 6.5±2. 3 | 42 | 9 |
| *Leptopsylla sicistae* | 4.5±1.5 | 27 | 2 |
| *Leptopsylla taschenbergi* | 6.8±2.1 | 25 | 5 |
| *Megabothris advenarius* | 6.5±1.8 | 39 | 6 |
| *Megabothris asio* | 4.0±0.0 | 19 | 2 |
| *Megabothris calcarifer* | 6.5±1.9 | 32 | 7 |
| *Megabothris rectangulatus* | 10.2±2.1 | 144 | 16 |
| *Megabothris turbidus* | 10.1±2.0 | 153 | 15 |
| *Megabothris walkeri* | 9.1±1.9 | 111 | 11 |
| *Mesopsylla eucta* | 6.7±0.7 | 37 | 3 |
| *Mesopsylla hebes* | 6.2±1.6 | 23 | 4 |
| *Mesopsylla lenis* | 8.0±1.7 | 23 | 3 |
| *Mesopsylla tuschkan* | 8.0±1.5 | 24 | 3 |
| *Myoxopsylla jordani* | 4.5±0.5 | 15 | 2 |
| *Neopsylla acanthina* | 4.8±1.1 | 20 | 5 |
| *Neopsylla bidentatiformis* | 6.0±1.7 | 22 | 4 |
| *Neopsylla democratica* | 3.0±0.0 | 14 | 2 |
| *Neopsylla galea* | 3.0±0.0 | 6 | 2 |
| *Neopsylla mana* | 12.4±4.0 | 87 | 11 |
| *Neopsylla meridiana* | 3.5±0.5 | 53 | 2 |
| *Neopsylla pleskei* | 8.9±2.8 | 66 | 11 |
| *Neopsylla setosa* | 7.4±2.6 | 65 | 7 |
| *Neopsylla teratura* | 7.0±1.8 | 44 | 5 |
| *Nosopsyllus aralis* | 6.3±1.4 | 24 | 3 |
| *Nosopsyllus consimilis* | 10.4±2.8 | 78 | 10 |
| *Nosopsyllus fidus* | 5.7±1.8 | 47 | 4 |
| *Nosopsyllus iranus* | 7.0±1.0 | 23 | 3 |
| *Nosopsyllus laeviceps* | 8.0±2.1 | 46 | 7 |
| *Nosopsyllus mokrzeckyi* | 6.7±2.2 | 33 | 4 |
| *Nosopsyllus tersus* | 5.0±1.0 | 22 | 3 |
| *Nosopsyllus turkmenicus* | 8.3±2.2 | 20 | 3 |
| *Ochotonobius hirticrus* | 5.0±1.2 | 16 | 2 |
| *Ophthalmopsylla kiritschenkovi* | 3.0±0.0 | 6 | 2 |
| *Ophthalmopsylla praefecta* | 3.0±0.0 | 3 | 1 |
| *Ophthalmopsylla volgensis* | 8.2±2.4 | 33 | 4 |
| *Oropsylla alaskensis* | 5.0±0.0 | 5 | 1 |
| *Oropsylla ilovaiskii* | 5.7±2.1 | 34 | 6 |
| *Oropsylla silantiewi* | 7.0±3.0 | 14 | 2 |
| *Palaeopsylla kohauti* | 3.5±0.5 | 7 | 2 |
| *Palaeopsylla soricis* | 10.6±2.1 | 181 | 17 |
| *Paradoxopsyllus dashidorzhii* | 6.7±1.7 | 20 | 3 |
| *Paradoxopsyllus hesperius* | 3.0±0.0 | 3 | 1 |
| *Paradoxopsyllus integer* | 4.0±0.0 | 8 | 2 |
| *Paradoxopsyllus repandus* | 5.0±0.0 | 10 | 2 |
| *Paradoxopsyllus scorodumovi* | 13.0±5.0 | 52 | 4 |
| *Paradoxopsyllus teretifrons* | 6.0±0.0 | 12 | 2 |
| *Paramonopsyllus scalonae* | 7.3±2.3 | 22 | 3 |
| *Paraneopsylla ioffi* | 5.0±1.3 | 20 | 4 |
| *Paraneopsylla tiflovi* | 3.5±0.5 | 7 | 2 |
| *Pectinoctenus nemorosa* | 5.2±1.3 | 26 | 5 |
| *Pectinoctenus pavlovskii* | 7.0±2.5 | 28 | 4 |
| *Pectinoctenus pectiniceps* | 5.7±1.2 | 17 | 3 |
| *Peromyscopsylla bidentata* | 7.2±1.8 | 93 | 13 |
| *Peromyscopsylla fallax* | 5.0±0.0 | 10 | 2 |
| *Peromyscopsylla ostsibirica* | 4.0±1.0 | 16 | 4 |
| *Peromyscopsylla silvatica* | 8.5±2.2 | 77 | 9 |
| *Rhadinopsylla altaica* | 5.5±0.5 | 11 | 2 |
| *Rhadinopsylla altifrons* | 3.0±0.0 | 6 | 2 |
| *Rhadinopsylla angusta* | 4.5±0.5 | 9 | 2 |
| *Rhadinopsylla bivirgis* | 3.5±0.5 | 7 | 2 |
| *Rhadinopsylla cedestis* | 8.5±2.8 | 34 | 4 |
| *Rhadinopsylla dahurica* | 7.0±2.5 | 21 | 3 |
| *Rhadinopsylla integella* | 8.2±2.6 | 49 | 6 |
| *Rhadinopsylla li* | 13.8±6.6 | 83 | 6 |
| *Rhadinopsylla pentacantha* | 3.7±0.3 | 11 | 3 |
| *Rhadinopsylla pseudodahurica* | 4.0±1.0 | 8 | 2 |
| *Rhadinopsylla rothschildi* | 7.0±0.0 | 7 | 1 |
| *Rhadinopsylla ucrainica* | 5.7±1.8 | 17 | 3 |
| *Rostropsylla daca* | 8.0±0.0 | 16 | 2 |
| *Stenoponia conspecta* | 4.0±0.0 | 8 | 2 |
| *Stenoponia sidimi* | 4.0±0.0 | 4 | 1 |
| *Stenoponia suknevi* | 4.0±1.0 | 8 | 2 |
| *Stenoponia tripectinata* | 3.5±0.5 | 7 | 2 |
| *Stenoponia vlasovi* | 7.7±1.4 | 23 | 3 |
| *Wagnerina tuvensis* | 4.0±0.0 | 8 | 2 |
| *Xenopsylla conformis* | 8.4±2.2 | 59 | 7 |
| *Xenopsylla gerbilli* | 11.7±1.2 | 35 | 3 |
| *Xenopsylla hirtipes* | 5.7±1.3 | 17 | 3 |
| *Xenopsylla nuttalli* | 7.0±0.0 | 14 | 2 |
| *Xenopsylla skrjabini* | 7.3±2.2 | 22 | 3 |

**Supplementary Table S2**

List of parasitic gamasid mite species that exploited at least three host species within a region

| Mite species | Mean (±S.E.) number of host species within a region | Total number of host species across regions | Number of regions where a mite species was recorded |
| --- | --- | --- | --- |
| *Androlaelaps angustiscutis* | 3.0±0.0 | 3 | 1 |
| *Androlaelaps casalis* | 6.2±1.9 | 25 | 4 |
| *Androlaelaps dogieli* | 7.5±0.5 | 15 | 2 |
| *Androlaelaps fahrenholzi* | 9.6±2.1 | 173 | 18 |
| *Androlaelaps pavlovskyi* | 5.0±1.0 | 10 | 2 |
| *Androlaelaps semidesertus* | 5.5±2.5 | 11 | 2 |
| *Eulaelaps cricetuli* | 5.5±0.5 | 11 | 2 |
| *Eulaelaps kolpakovae* | 4.0±0.0 | 8 | 2 |
| *Eulaelaps stabularis* | 10.1±2.1 | 223 | 22 |
| *Haemogamasus ambulans* | 9.7±1.8 | 223 | 23 |
| *Haemogamasus dauricus* | 7.2±2.1 | 29 | 4 |
| *Haemogamasus hirsutosimilis* | 7.5±0.5 | 15 | 2 |
| *Haemogamasus hirsutus* | 5.4±1.7 | 27 | 5 |
| *Haemogamasus horridus* | 6.3±2.4 | 19 | 3 |
| *Haemogamasus ivanovi* | 3.0±0.0 | 6 | 2 |
| *Haemogamasus liponyssoides* | 6.8±2.4 | 41 | 6 |
| *Haemogamasus mandschuricus* | 8.4±2.5 | 59 | 6 |
| *Haemogamasus nidi* | 8.8±1.8 | 149 | 17 |
| *Haemogamasus nidiformis* | 6.4±1.5 | 77 | 12 |
| *Haemogamasus serdjukovae* | 5.8±1.6 | 29 | 5 |
| *Hirstionyssus apodemi* | 11.3±3.41 | 68 | 6 |
| *Hirstionyssus carnifex* | 3.0±0.0 | 3 | 1 |
| *Hirstionyssus criceti* | 7.2±2.9 | 29 | 4 |
| *Hirstionyssus eusoricis* | 8.9±1.9 | 129 | 14 |
| *Hirstionyssus gudauricus* | 4.5±0.5 | 9 | 2 |
| *Hirstionyssus isabellinus* | 9.8±1.8 | 241 | 24 |
| *Hirstionyssus latiscutatus* | 6.7±2.0 | 20 | 3 |
| *Hirstionyssus transiliensis* | 6.7±1.4 | 27 | 4 |
| *Hyperlaelaps amphibius* | 6.0±1.6 | 31 | 5 |
| *Hyperlaelaps arvalis* | 7.0±1.6 | 95 | 14 |
| *Hyperlaelaps microti* | 9.0±1.5 | 27 | 3 |
| *Laelaps agilis* | 7.0±1.6 | 42 | 6 |
| *Laelaps alaskensis* | 3.0±0.0 | 6 | 2 |
| *Laelaps algericus* | 5.2±1.3 | 21 | 4 |
| *Laelaps clethrionomydis* | 7.9±1.6 | 151 | 19 |
| *Laelaps hilaris* | 7.6±1.5 | 107 | 14 |
| *Laelaps lemmi* | 5.0±1.7 | 20 | 4 |
| *Laelaps micromydis* | 5.0±2.0 | 10 | 2 |
| *Laelaps multispinosus* | 5.5±1.9 | 22 | 4 |
| *Laelaps muris* | 7.9±2.0 | 71 | 9 |
| *Laelaps nuttalli* | 8.2±2.2 | 33 | 4 |
| *Laelaps pavlovskyi* | 9.5±2.2 | 95 | 10 |
| *Myonyssus decumani* | 3.0±0.0 | 3 | 1 |
| *Myonyssus dubinini* | 5.5±0.5 | 11 | 2 |
| *Myonyssus gigas* | 5.0±0.0 | 10 | 2 |
| *Myonyssus ingricus* | 6.5±1.9 | 26 | 4 |
| *Myonyssus rossicus* | 3.0±0.0 | 3 | 1 |
